# 53746 Body Composition and Metabolic Profiles in Infants of Diabetic Mothers (IDM) as Predictors of Hunger Signaling Gene Expression

**DOI:** 10.1017/cts.2021.756

**Published:** 2021-03-30

**Authors:** Dara Azuma, Jill Maron

**Affiliations:** 1 Tufts Medical Center; 2 Mother Infant Research Institute

## Abstract

ABSTRACT IMPACT: This study aims to advance the understanding of the biological mechanisms associated with feeding disturbances in infants born to diabetic mothers through non-invasive salivary gene expression analyses and body composition measurements at birth. OBJECTIVES/GOALS: To determine if non-invasive salivary gene expression analyses and body composition measurements at birth could elucidate biological mechanisms associated with aberrant feeding behaviors and disrupted metabolic profiles commonly seen in infants born to diabetic mothers. METHODS/STUDY POPULATION: This prospective cohort study enrolls subjects born at ≥35 weeks’ gestation without a history of intrauterine growth restriction or major congenital anomalies. The diabetic cohort is defined as infants born to mothers with gestational diabetes or type 2 diabetes. The primary outcome is salivary expression of the hunger signaling genes, AMPK and NPY2R. Secondary outcomes include infant body composition measurements, obtained by skinfold measurement and/or air displacement plethysmography, and salivary expression of the adipokines, leptin, ghrelin, and adiponectin. Multiple logistic regression will be used to determine which factors are associated with AMPK and NPY2R expression. RESULTS/ANTICIPATED RESULTS: We propose that poor oral intake seen in infants of diabetic mothers may be due to alterations in the expression of hunger signaling genes (decreased expression of AMPK; increased expression of NPY2R) resulting in a diminished feeding drive in these large for gestational age infants. In addition, infant adiposity and the expression of genes involved in the adipoinsular axis will be inversely proportional to feeding volume intake. Namely, increased neonatal fat mass will be associated with increased expression of leptin and decreased expression of ghrelin and adiponectin. DISCUSSION/SIGNIFICANCE OF FINDINGS: Infants of diabetic mothers are at higher risk of poor oral feeding in the newborn period. This study aims to elucidate the link between neonatal body composition, adipoinsular axis, and hunger signaling to explain this unique feeding phenotype.

